# Optical beaming of electrical discharges

**DOI:** 10.1038/s41467-020-19183-0

**Published:** 2020-10-20

**Authors:** V. Shvedov, E. Pivnev, A. R. Davoyan, W. Krolikowski, A. E. Miroshnichenko

**Affiliations:** 1 Laser Physics Centre, Research School of Physics, Australian National University, Canberra, Australia; 2 Science Program, Texas A&M University at Qatar, Doha, Qatar; 3 Department of Mechanical and Aerospace Engineering, University of California, Los Angeles, USA; 4 School of Engineering and Information Technology, University of New South Wales, Canberra, Australia

**Keywords:** Applied optics, Optical manipulation and tweezers

## Abstract

Igniting and guiding electrical discharges to desired targets in the ambient atmosphere have been a subject of intense research efforts for decades. Ability to control discharge and its propagation can pave the way to a broad range of applications from nanofabrication and plasma medicine to monitoring of atmospheric pollution and, ultimately, taming lightning strikes. Numerous experiments utilizing powerful pulsed lasers with peak-intensity above air photoionization and photo-dissociation have demonstrated excitation and confinement of plasma tracks in the wakes of laser field. Here, we propose and demonstrate an efficient approach for triggering, trapping and guiding electrical discharges in air. It is based on the use of a low-power continuous-wave vortex beam that traps and transports light-absorbing particles in mid-air. We demonstrate a 30% decrease in discharge threshold mediated by optically trapped graphene microparticles with the use of a laser beam of a few hundred milliwatts of power. Our demonstration may pave the way to guiding electrical discharges along arbitrary paths.

## Abstract (web summary)

Control of electrical discharge paths would allow several technological applications, but it usually requires air photoionisation with high-peak-power pulsed lasers. Here, instead, the authors exploit the trapping and heating of light-absorbing particles to guide discharge along the desired path.

## Introduction

Electrical discharge in gases is manifested in a wide range of high voltage systems^1^ and natural lightning phenomena^2^. Plasma discharges play a vital role in materials deposition and fabrication techniques^3,4^—the backbone of nanotechnology and semiconductor electronics. High voltage discharges are of fundamental significance for accelerator physics and high energy photon sources^5–7^. Low power atmospheric discharges, in turn, have recently emerged as a robust plasma medicine tool for cancer treatment^8–12^. While the physics of electrical discharge and spark formation is understood in great detail^1,13^, efficient control of ultrafast avalanche breakdown processes and subsequent guiding of discharges along predictable paths in ambient air constitute a significant challenge^14^.

Proliferation of laser technology offers new frontiers in triggering and guiding of electrical discharges^15–27^. In particular, pulsed laser beams with electric field intensities above air photoionization threshold were shown to induce plasma channels in which electrical discharge could be sustained and guided^20–27^. Such direct optical field-induced photoionization requires very high optical field intensities, comparable with the atomic binding field (~10^11^ to 10^12^ V m^−1^). Use of such high peak-power laser beams may limit the scope of applications. At the same time, to the best of our knowledge, no techniques that can control electrical discharges with low power laser beams exist.

Here, we demonstrate a conceptually different approach to inducing and guiding electrical discharge along predetermined paths to desired targets over long distances in air. Our approach does not rely on photoionization. Instead, we locally control the mean free path of electrons in the ambient air and tailor conditions of electric breakdown.

## Results

### Laser-induced air breakdown with optical trapping mechanism

**Fig. 1:**
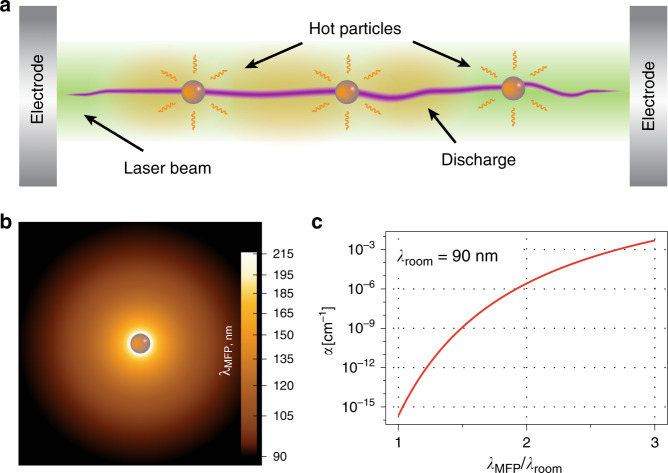
Mechanisms for a laser-induced air breakdown and discharge guidance. Laser vortex beam that traps particles and at the same time heats them (**a**) leading to the local reduction in the air density and the increase of the electron mean free path *λ*_MFP_ in heated regions surrounding the particles (**b**). This dramatically increases the Townsend ionisation coefficient *α* (**c**) in hot areas resulting in a decrease of the discharge threshold.

The key to our technique is the use of a long-range optical manipulation—a beam of light that guides light-absorbing particles in ambient air^28–31^, see Fig. 1a. Specifically, by trapping, placing and heating these particles with the laser beam in mid-air, we locally increase the air temperature and modify the breakdown conditions, Fig. 1b, c, enabling discharge over sustained predefined paths. The only requirement for our technique is the conversion of laser radiation into the heat of trapped particles, implying that the process is agile to the choice of particle materials. Hence, our method works equally well with metals and dielectrics. Our approach is fundamentally different from previously employed high light intensity and pulsed laser techniques (see Supplementary Fig. 1) and provides new avenues to explore its potential for higher efficiency and application in longer range systems.

In order to elucidate the physical principles of our approach, we start with a simple model scenario of a single particle located between two parallel electrodes in air. Air breakdown and discharge formation are associated with avalanche ionisation and may be described by the following breakdown condition^20^:

1 $$1\,=\,\gamma e^{\alpha l},$$

here *γ* is a constant specific to a given setup, *l* is the distance between capacitor plates, and $\alpha = \frac{1}{{\lambda _{\mathrm{e}}}}\exp \left( { - \frac{l}{{\lambda _{\mathrm{e}}}}\frac{{{\cal{E}}_{\mathrm{i}}}}{{e{{V}}_{\mathrm{b}}}}} \right)$ is the Townsend ionisation coefficient, *λ*_e_ is the electron mean free path, ${\cal{E}}_{\mathrm{i}}$ is the energy of ionisation; *V*_b_ is the breakdown voltage sought for, and *e* is the elementary charge. It turns out that the Townsend ionisation coefficient *α* strongly depends on the electron mean free path *λ*_e_, as indicated in Fig. 1c. In particular, if the mean free path is increased by a factor of three (compared to the standard conditions) the ionisation coefficient *α* varies by 12 orders of the magnitude (see Supplementary Note 1 for detailes). Hence, by modifying the electron mean free path, *λ*_e_, for instance, by varying gas pressure or temperature, the ionisation coefficient can be strongly modified, and, thus, the breakdown condition, *V*_b_/*l*. Indeed, larger mean free path implies that electrons gain higher kinetic energy between collisions, leading to a dramatic increase in ionisation probability.

### Electrical discharge induced by a heated microspere

**Fig. 2:**
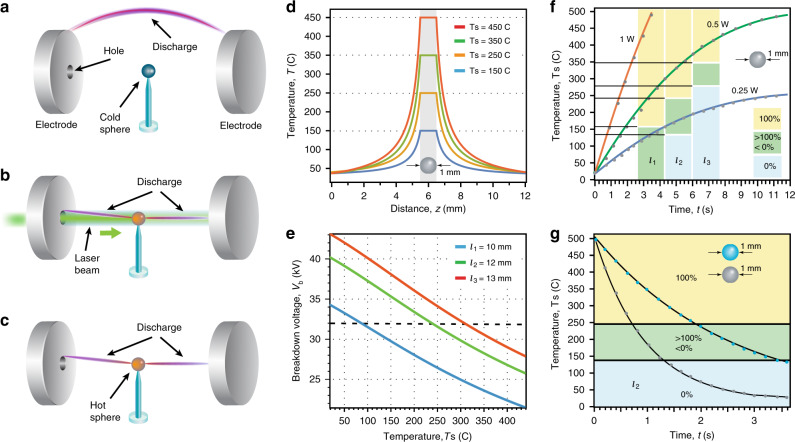
The electrical discharge induced by a heated microparticle. A single microparticle is fixed between two parallel electrodes energised close to the threshold breakdown of air. When the particle is cold, the electrical breakdown may occur along random trajectories, typically arcing close to the edges of the electrodes (**a**). Heating the sphere with a laser beam results in guiding the discharge through the sphere (**b**). The heated sphere attracts the electrical spark even after the laser is turned off (**c**). Theoretical estimation (**d**) shows the temperature distribution around the heated sphere of 1 mm diameter (Supplementary Note 2). Theoretical estimation (**e**) shows the breakdown voltage threshold for the fixed distances between the electrodes depending on the sphere temperature in the range of our experimental conditions (For the higher temperature scale see Supplementary Fig. 3). Dashed line in **e** denotes DC voltage applied in our experiment (*V* = 32 kV). Experimental graphs (**f**, **g**) detail the phenomenon as the measured temperature of a 1-mm metallic (grey) and glass (blue) sphere versus time of heating by the laser of three different powers (**f**) or cooling when the laser is of (**g**). The breakdown probabilities between the electrodes are shown in blue, green, and yellow colours, for the voltage of 32 kV and the interelectrode separation of *l*_1_ = 10 mm, *l*_2_ = 12 mm, and *l*_1_ = 13 mm. Data points are shown with circles; the line is a numerically calculated dynamics of the surface temperature variation.

For the simplest illustration of our principle, we first consider a single, 1 mm diameter, sphere placed between two plane electrodes with 10 mm long (or larger) interelectrode gap, as shown in Fig. 2a–c. Because of its size (1 mm diameter particle inside 10 mm long (and larger) interelectrode gap), we may safely assume that the particle is too small to modify electric potential between the plates. As long as the laser beam is off, the entire system behaves as a regular air-gap parallel-plate capacitor. At these conditions, the air breakdown and formation of discharge arcs are expected, when the applied voltage is above a threshold value for a given electrode separation distance (i.e., *V* ≥ *V*_b_ at given *l*)^1^.

Upon laser illumination, the particle is heated via light absorption, and the discharge is expected for the applied voltage below the threshold value for the same interelectrode distance (Fig. 2b). Similarly, the discharge will take place even after the laser beam is blocked, as long as the particle is still hot (Fig. 2c). In a steady-state, the temperature of the particle depends on the laser beam power, particle absorptivity, and heat conduction properties at the interfaces. Figure 2d shows a typical temperature profile in the particle and in the surrounding air. The local change of temperature of the surrounding air changes its density, and, respectively, the electron mean free path (see also Fig. 1c):

2 $$\lambda _{\mathrm{e}}(x) = \frac{{k_{\mathrm{B}}{\mathrm{T}}\left( x \right)}}{{\sigma _{\mathrm{i}}{\mathrm{p}}}},$$

here, *k*_B_ is the Boltzmann constant, *σ*_i_ is the ionisation cross-section, and *p* is the ambient pressure. The higher average temperature in the channel and, respectively, higher mean free path imply that favourable ionisation pathways exist, and overall lead to a lower breakdown threshold voltage. Figure 2e shows the calculated breakdown voltage with the variation of the particle temperature. This simple model predicts >20% reduction of the breakdown voltage for a particle heated to 200 °C, i.e., 28 kV for a 10 mm gap. That is, local temperature variations create a channel with a favourable path for a discharge to occur. Indeed, air regions with higher temperature correspond to longer electron mean travel distances between collisions leading to a higher probability of air ionisation in those regions (Fig. 1c). By controlling the temperature distribution, e.g., by placing several light-absorbing particles, a predetermined discharge path in the air may be created. Notably, the particle only plays a role of breakdown mediator via laser heating, its material—be it dielectric or metal—plays no role in gas dynamics or avalanche processes.

In order to quantify the scenario sketched above, we perform a series of experiments with both electrically conductive (stainless steel sphere coated with 300 nm of carbon) and dielectric (dark glass) light-absorbing spheres. The spheres are 1 mm in diameter and are fixed on a glass post at the middle point between the two plate electrodes biased with a DC voltage of 32 kV. Next, we set the electrode separation distance at one of the three fixed values of 10, 12, and 13 mm and study the discharge formation as a function of the particle temperature under continuous-wave laser (Verdi V5 @532 nm) illumination. To this end, we measure the temporal variation of the sphere’s temperature for several values of the laser power (Fig. 2f), while simultaneously recording the discharge processes with a CCD camera (see “Methods” section). We denote the observed probability of breakdown with a stacked bar chart in Fig. 2f. Initially, when the laser is off, the sphere temperature is that of the ambient air, i.e., ∼22 °C. For a 10 mm interelectrode gap, at this condition, discrete discharges are observed only close to the edges of the electrodes, as sketched in Fig. 2a (see Supplementary Movie 1 and Supplementary Movie 2). No discharge is observed, as expected, for 12 and 13 mm gaps, since the applied voltage is well below the air breakdown threshold for those gaps. Upon laser illumination, as the temperature of the sphere reaches ∼150 °C, we observe that the air breakdown condition is reached with clear discharge passing through the sphere for both 10 and 12 mm gaps, as shown schematically in Fig. 2b (see Supplementary Movie 1 and Supplementary Movie 2). The discharge still does not occur for the 13 mm gap between the electrodes. With a further increase of the sphere’s temperature above 350 °C the discharge does occurs and passes only through the sphere for all three gap lengths (see Fig. 2f and Supplementary Movie 3). This behavior closely matches our theoretical prediction of the breakdown condition (see Fig. 2e).

To elucidate the role of laser in the discharge, we measure the discharge probability after the laser is turned off while the sphere is still hot (see Fig. 2c, g). The discharge is always observed when the sphere’s temperature is above the threshold value (Fig. 2g), which is also predicted by our model (Fig. 2e). At high temperatures discharge is independent of laser illumination (i.e., on or off) or material composition of the sphere. Hence, identical results for both metallic (Supplementary Movie 4) and dielectric (Supplementary Movie 5) spheres were attained indicating that the discharge does not depend on the electrical properties of the particle. We conclude that both thermoionic emission and photoionization processes are not manifested in the discharge formation.

We stress that the suggested approach to discharge guidance along the path of elevated temperature regions differs drastically from previously demonstrated high-intensity and pulsed laser techniques, Fig. 1a, b. Specifically, it was shown that with the use of high peak-power pulsed lasers gas ionisation along the path of the laser beam might be induced. Direct laser-plasma interaction and the need for energetic short pulses limit the efficiency and the propagation range. Our technique does not utilise direct laser-driven air ionisation and hence does not suffer from these limitations. Besides, our method operates under continuous-wave illumination and can be performed with ~mW power levels, i.e., orders of magnitude smaller than previous demonstrations.

### Optical trapping for discharge control and guidance

**Fig. 3:**
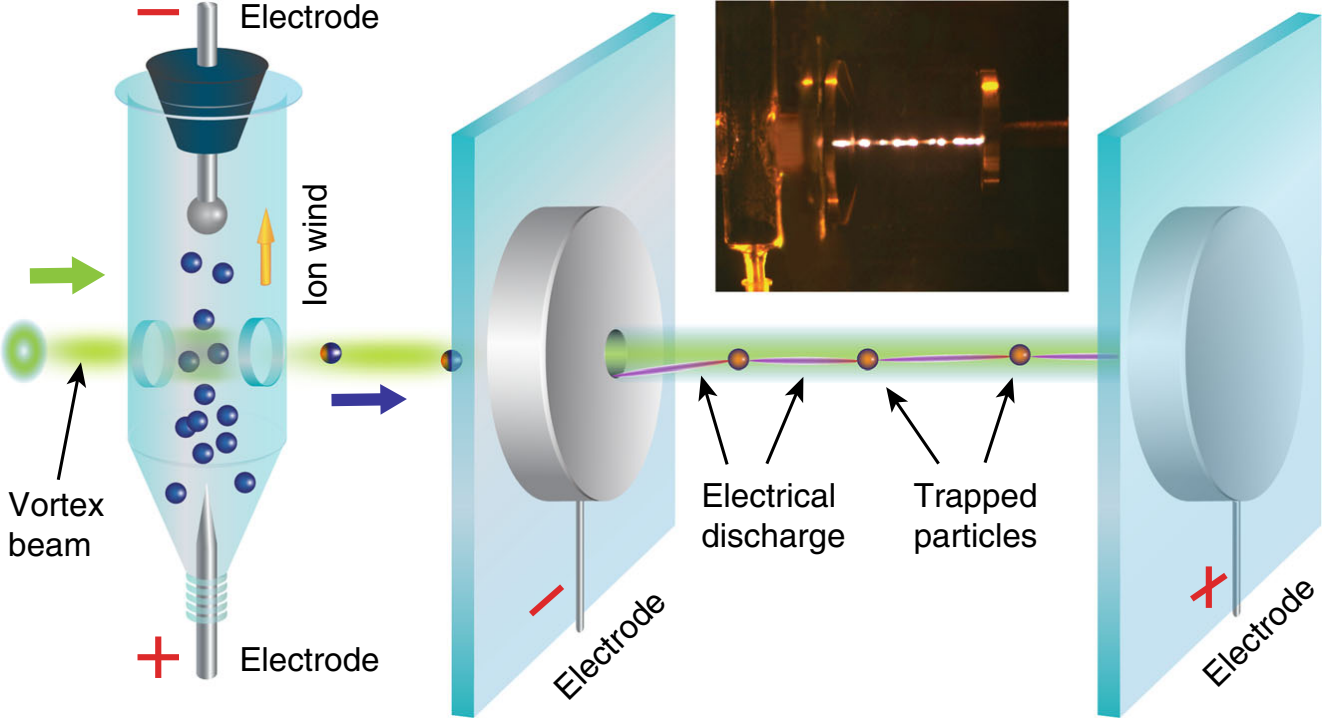
Experimental configuration and principles of optical vortex beam for discharge control and guidance. At first, microparticles are prepared and placed in an enclosed container with vertically aligned electrodes (left). An applied electric field through the container lifts the particles by electrostatic forces. Some of the lifted particles cross the hollow core doughnut-shaped CW laser beam and get trapped in it. The vortex laser beam is guides particles in the mid-air: particles from a container are transported into a parallel plate chamber through a hole in one of the electrodes. In addition, particles are heated by the same laser beam thus creating a thermal channel between the electrodes that results in a subthreshold electrical breakdown. The inset shows a photograph of the experimental setup with particles trapped in the beam. Channelling of the discharge along the beam path is clearly observed (Supplementary Movie 6).

Next, we show that the discharge may be guided along predetermined paths with particles suspended in mid-air. For this purpose, we employ vortex beam optical trapping^28–30^. Figure 3 illustrates the experimental configuration of our setup for a laser-driven breakdown in air. In particular, we deliver light-absorbing graphene particles (100–1000 μm in size) to the space between the electrodes using a slowly diverging laser vortex beam (see “Methods” section) propagating through a round hole in the first electrode. Here, the laser beam plays two simultaneous roles: it acts as a particle conveyor and their heat source. With sufficient laser power, the particle temperature rises above the threshold value triggering the electric breakdown (Fig. 3 inset). Here, we make use of particles made of graphene, which are light weight, highly absorbing, and can withstand high temperatures. However, our concept can be extended to particles made of other materials as well (e.g., high temperature and low sputtering yield materials capable of withstanding repeated discharges). As well as particles deliberately engineered for efficient trapping^30^. In our experiments, the particles are transported along the beam propagation direction. However, one can design an optical setup where particles are delivered over the long range and stably positioned at certain location in mid-air by optical beams^30^.

**Fig. 4:**
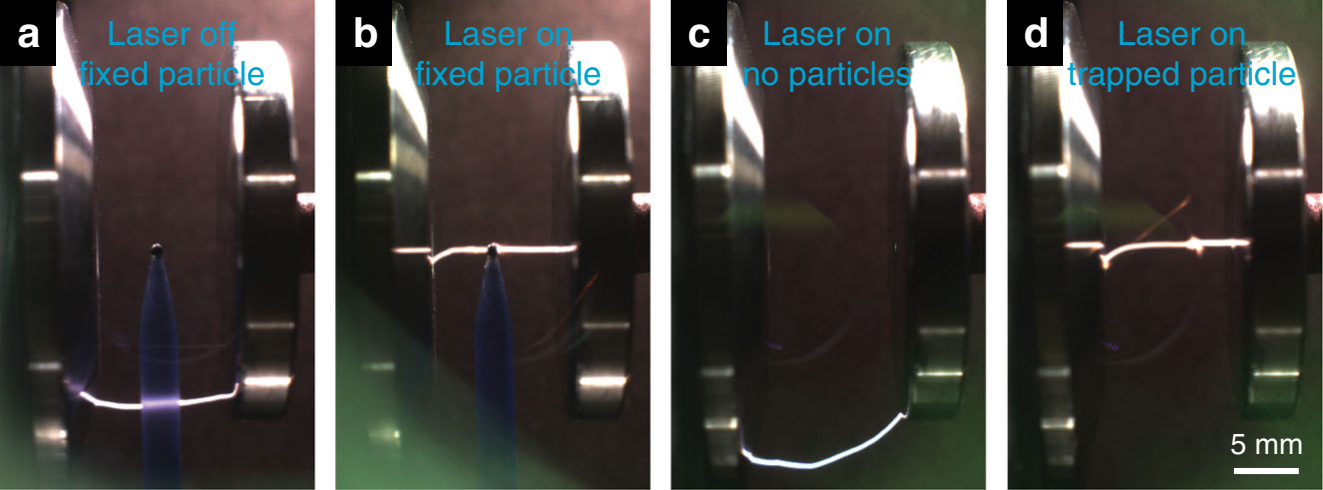
Experimental study of the breakdown formation and discharge guidance. **a**, **b** CCD micrographs elucidating the role of a fixed particle and its laser heating in the breakdown and discharge guidance: without any illumination at near-breakdown conditions the discharge follows random paths (**a**), whereas upon illumination (**b**) discharge is always guided through the particle. **c**, **d** Show the operation of a vortex transporter beam. With no particles trapped in the beam (**c**) discharge is not affected by the laser beam (cf. panel **a**). However, when particles are trapped in the vortex beam and are brought between the electrodes, discharge always passes through the particles. In all of these cases, the voltage is 32 kV and the distance between electrodes is 10 mm, i.e., close to air-breakdown at normal conditions. A 532 nm CW laser with 500 mW power is used.

Figure 4 shows CCD images of several of conducted experiments that demonstrate transporter beam operation and reveal the role of the laser illumination in discharge formation. Here, a distance between electrodes is fixed at 10 mm, and the applied voltage is 32 kV, i.e., close to a breakdown at the normal conditions. For a particle fixed on a post electrical discharge occurs predominantly at the electrode edges, where the electrical field is maximal (Fig. 4a), as is expected for a case with no illumination, see also Fig. 2a. Evidently, the particle has no role in the discharge formation or guidance. Upon particle illumination with a laser, discharge passes directly through this particle (Fig. 4b), according to the physics detailed earlier. With no particles inserted between the electrodes (Fig. 4c and Supplementary Movie 7) the discharge dynamics is that of a regular parallel plate capacitor and is independent of the laser field, cf. Fig. 4a. However, when the vortex beam traps and delivers particles into the interelectrode space, the electrical discharge always follows a trajectory through the particles (Fig. 4d and Supplementary Movie 8). This scenario generally repeats the discharge dynamics in the case of a fixed illuminated particle described earlier (Fig. 4b). However, in contrast to a fixed particle case, our vortex beam provides remote delivery of the particles to the desired location, thus enabling precise control of the electrical discharge pathway in mid-air. Notably, our experimental setup allows transporting particles only along the direction of the beam propagation. Therefore, to create a sustained discharge pathway continuous resupply of particles is needed. However, the optical delivery system can be modified to stably trap and position particles in a desired location, thus enabling an optically reconfigurable discharge pathway.

**Fig. 5:**
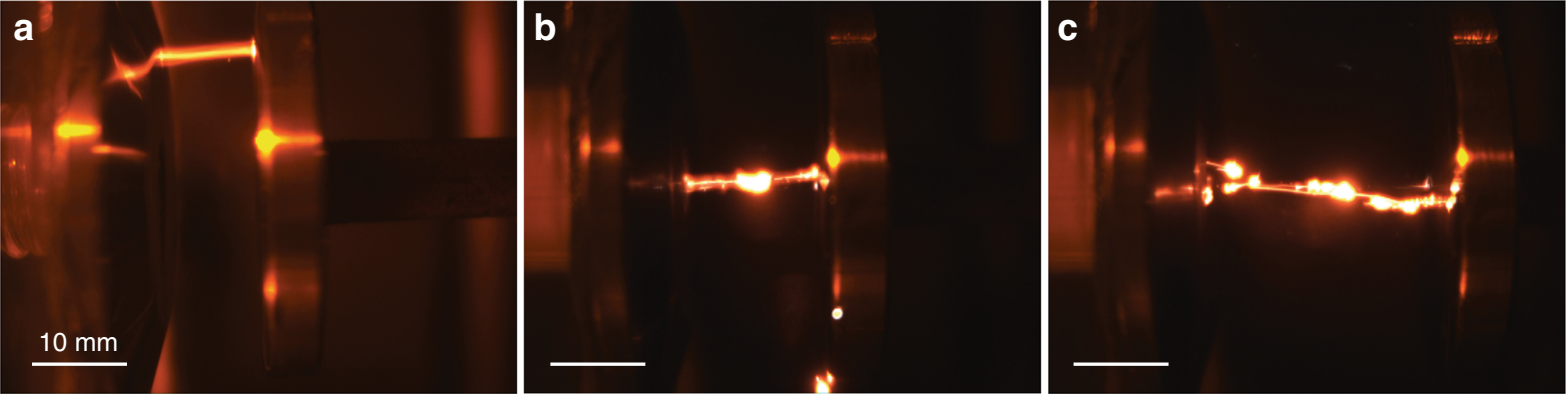
Vortex beam guided electrical discharge with multiple trapped particles. **a** Demonstrate an electrical breakdown at standard conditions (32 kV and 10 mm distance) without trapped particles. **b**, **c** Demonstrate an observation of a guided electrical discharge with multiple trapped particles for 15 mm and 25 mm air gaps, respectively.

By trapping multiple particles spaced closely to each other an additional possibility to create favourable discharge channels is created (see Supplementary Fig. 2 and Supplementary Note 3). In this case discharge is guided along desired paths predetermined by position of the trapped particles. Such channels may be substantially longer than the maximum electrode separation distance, *l*_max_, for a given threshold voltage, *V*_b_, under normal conditions. Figure 5a–c detail a corresponding experimental demonstration. Without particles trapped in the laser beam, discharge is observed at standard breakdown conditions: in our case for an applied voltage of *V*_b_ = 32 kV this corresponds to a maximum electrode separation distance of *l*_max_ = 10 mm. For larger air gap separation (i.e., *l* > *l*_max_) no breakdown is possible. The trapping of multiple particles along the laser channel alters breakdown condition by creating a longer conduction path for discharge guidance. Figure 5b, c show breakdown and discharge formation for 15 mm and 25 mm electrode separation distances, respectively, that is, one and a half and two and a half times the maximum distance at normal conditions (Supplementary Movie 9 and Supplementary Movie 10).

## Discussion

In summary, our experiments and theoretical predictions demonstrate that with an optical beam guiding trapped particles the threshold conditions for an electrical breakdown may be significantly decreased, and discharge may be guided along a predetermined path in the air. Here, we have used a single vortex transporter beam and shown guidance along a straight line. However, our approach is applicable to more complex scenarios. Notably, we envisage guidance of electrical discharges along complex three-dimensional paths in the ambient air with the use of multiple and/or spatially modulated beams including beams propagating along curved trajectory, such as Airy vortex beams^31,32^. In addition, the technique we propose pertains to atmospheres with a different gas composition and/or pressure level. Furthermore, low power continuous wave operation ensures ease of implementation and paves the way for a range of applications such as controlling high voltage systems^1^, microfabrication and machining^3,4^, precision microsurgery and cancer treatment^8–12^, and controlling atmospheric lightning^2,33^.

## Methods

### Vortex transporter beam preparation

In our experiments, we used TEM01 laser beam with a doughnut-shaped intensity distribution. The intensity of such a beam can be represented as:

3 $$I(\rho ,z)\,=\,I_0(\rho ^2/{\mathrm{w}}^2){\mathrm{exp}}( - \rho ^2/{\mathrm{w}}^2),$$

where *I*_0_ denotes the maximal intensity, *ρ* is the radial coordinate measured from the beam axis, *z* is an axial coordinate along the beam axis, *w*^2^ = *w*_0_^2^(1 + *z*^2^*λ*^2^ /(4*π*^2^*w*0^4^) is the beam waist with propagation, *w*_0_ is the initial waist (at *z* = 0), and *λ* is a wavelength of the CW laser beam.

Such a beam was prepared with the use of standard polarisation conversion of circularly polarised Gaussian beam^30,34^. Specifically, a beam from a CW laser (*λ* = 532 nm) passed through a 2-mm-long biaxial KTP crystal placed between two identical microscope objectives (NA = 0.25). One of the optical axes of the crystal was aligned with the beam propagation direction. This led to conical refraction of light and to the formation of the beam with a complex polarisation structure. After passing through a quarter-wave plate and a polariser, the beam acquired a doughnut-shaped intensity distribution and singular phase profile (i.e., topologically charged optical vortex). The beam waist, *w*_0_, could be varied by changing the distance between the microscope objectives. In our experimental setup, we chose *w*_0_ = 0.5 mm, which ensures a very low divergence of the beam with a nearly uniform intensity profile through the entire experimental setup (see Fig. 1).

The hollow core beam was used to trap and deliver the particles as is detailed in the text. As the particles reached their designated position between the electrodes, we applied 32 kV to the main electrodes so that electrical wind^35^ prevented their motion in the opposite direction. This allowed one to stabilise particle motion along the beam and avoid reaching the electrode before the discharge. Note that polarity of the electrodes is not critical here.

### Temperature measurements

The temperature of steel and dielectric spheres fixed on the post, as well as that of trapped particles, was measured with a CCD camera which was first calibrated using a thermocouple. To this end, we first attached a metallic sphere to the pin of thermocouple and placed it in the exact position of the laser beam, where the trapping has occurred. Then we measured the sphere temperature in that position for diffrent intensities of the laser beam. At the same time the high resolution CCD (operating in grey scale mode) attached to the microscope took series of pictures of the sphere in the IR regime. The light from the sphere that reached the CCD matrix, was passing the notch filter, NF, that cut off all the visible laser radiation (532 nm). Then, comparing the actual tempetrature measured with thermocouple with the intensity of emitted IR light as recorded by the CCD, we obtained the temperature scale, which was used to determine the temperature of particles trapped and transported by the light beam.

### Breakdown probability measurements

To determine the probability of the electrical discharge in the presence of hot particles, we measured temperature of the discharge event that occurs through a 1-mm metallic or 1-mm glass spheres. The spheres fixed between the electrodes with applied voltage, were heated by the laser beam of different powers or cooled when the laser beam was blocked. When heated, the temperature of the spheres was gradually increasing depending on the laser power and time of illumination, until it reached the breakdown threshold for the given interelectrode separation. In versa, when the laser beam was blocked, the spheres were gradually cooling down, and the discharge disappeared when the temperature of the spheres reached certain value. We conducted the same experiment 20 times for each sphere in heating/cooling regimes, and recorded the observations for the same initial conditions (i.e., voltage and gap separation distance). The results of these measurements were then used to prepare histograms of the discharge probability depicted in Fig. 2f, g.

## Supplementary information

Supplementary Information

Description of Additional Supplementary Files

Supplementary Movie 1

Supplementary Movie 2

Supplementary Movie 3

Supplementary Movie 4

Supplementary Movie 5

Supplementary Movie 6

Supplementary Movie 7

Supplementary Movie 8

Supplementary Movie 9

Supplementary Movie 10

## Data Availability

The authors declare that the data supporting the findings of this study are available within the paper. All relevant additional data are available from the corresponding author upon reasonable request.
