# Supplementary material for: Optical beaming of electrical discharges
Source: Nat Commun. 2020 Oct 20;11:5306. doi: 10.1038/s41467-020-19183-0 (PMC7576779; doi:10.1038/s41467-020-19183-0)
Supplement: Supplementary file 1 — Supplementary Information [file 41467_2020_19183_MOESM1_ESM.pdf]

Supplementary Information

Optical beaming of electrical discharges

Shvedov et al.

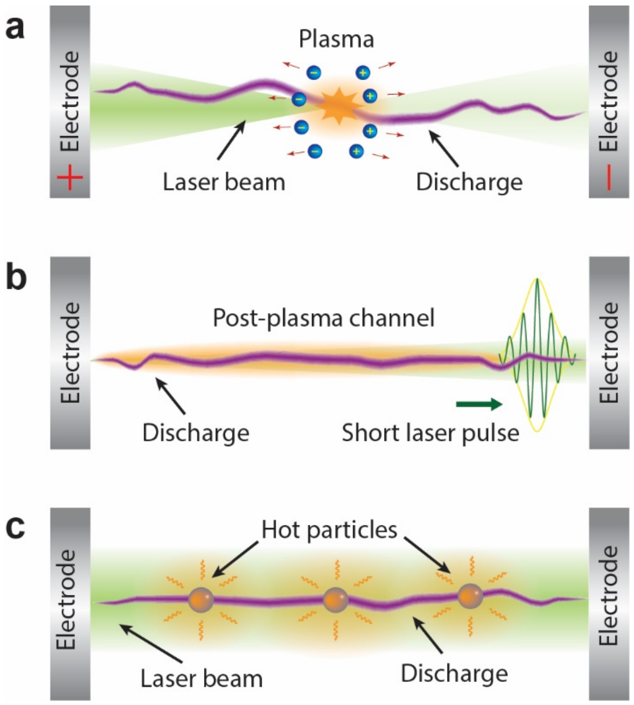

Supplementary Figure 1. **Mechanisms for a laser-induced air breakdown and discharge guidance.** Under laser illumination air break-down may be induced: (a) via direct air photoionisation with focused high-intensity laser beam[1,2,3,4,5,6,7,8] or (b) by the creation of low-pressure post-plasma channels with ultrafast laser pulses[9,10,11,12,13]. In contrast, our technique, schematically shown in (c), makes use of a low-power continuous-wave laser beam– a tractor beam, that traps and heats particles resulting in a decrease of the discharge threshold in hot air around the particles.

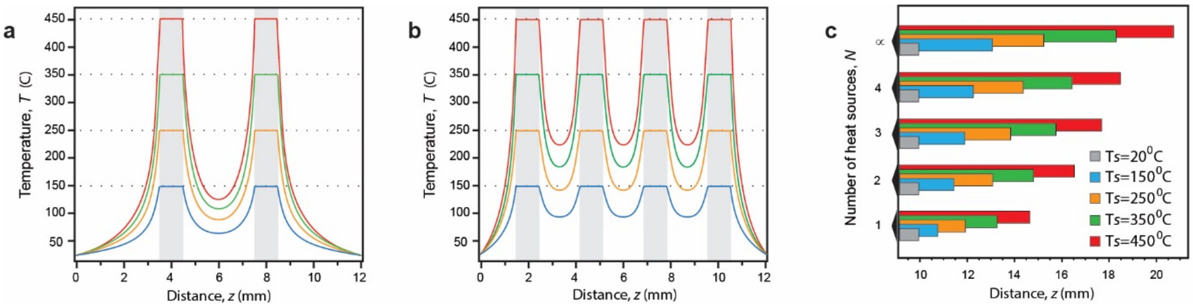

Supplementary Figure 2. **Calculated conditions for electrical discharge with multiple heated spherical particles of 1mm diameter.** Graphs (d), (e) show the temperature distribution in the air along the chain of the heated to 450 C particles for 4 mm and 2.5 mm between the particles, respectively. (f) The calculated maximum interelectrode separation distance for 32 kV of applied voltage with several infinitesimal heat sources at different temperatures.

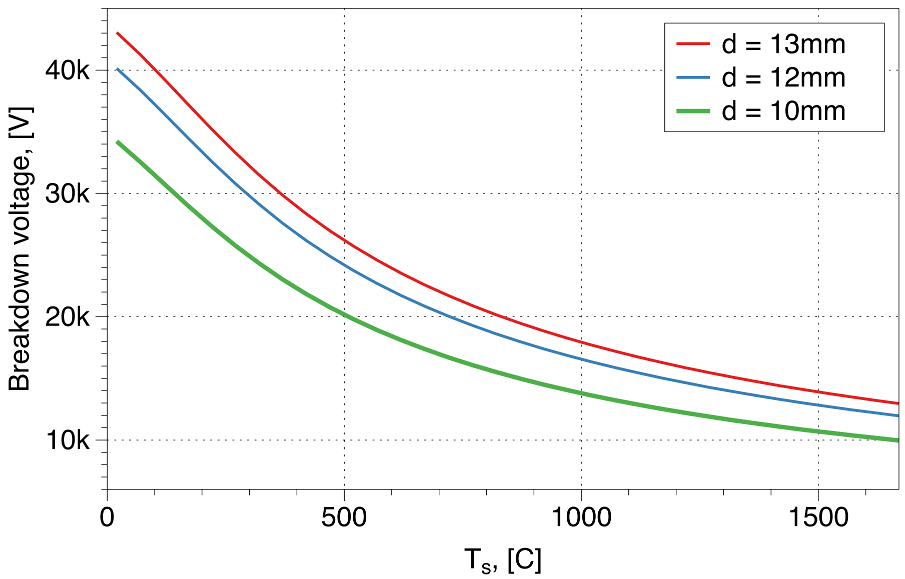

Supplementary Figure 3. **Calculated breakdown voltage for a single particle heated up to 2000K.** These results extend Fig.2(e) of the main manuscript and demonstrate that the breakdown voltage can be reduced by more than 3 times. The particle surface temperature changes from room up to 2000K, corresponding to the thermal ionization threshold.

Supplementary Note 1. **THEORETICAL MODEL FOR CALCULATION OF THE MODIFIED BREAKDOWN VOLTAGE**

The discharge between two electrodes under normal conditions is can be described by Paschen's law that was determined empirically and later explained by Townsend. Specifically, Townsend studied exponential current increase due to electron avalanche and showed that this current can be self-sustained in the channel (i.e., a breakdown condition) if the following certain criteria is met:

$$\gamma(e^{\alpha d} - 1) = 1, \tag{1}$$

where α is the so-called first Townsend coefficient that describes electron avalanche (i.e., exponential current increase, $I = I_0 e^{\alpha d}$), d is the distance between electrodes, γ is the so-called second Townsend coefficient that relates physically describes probability of secondary electron emission from the electrodes. Since usually $e^{\alpha d} \gg 1$ the Townsend breakdown condition can be simplified to

$$\gamma e^{\alpha d} \simeq 1 \tag{2}$$

By definition the first Townsend coefficient, α, describes number of ionisations per length of path. It can be presented as a product of number of collisions per unit length and the ionization probability:

$$\alpha = \frac{1}{\lambda_e} e^{-E_i/E_e}, \tag{3}$$

where $\frac{1}{\lambda_e}$ corresponds to the number of collisions per unit length and is related to the electron mean free path λ_e; and e^{-E_i/E_e} is the probability of ionization, i.e., the probability of an electron energy $E_e = e\lambda_e\mathcal{E}$ exceeds energy of ionization E_i; here e – elementary charge, and $\mathcal{E} = V/d$ is the electric field between the electrodes, V is the applied voltage. It allows to express the first Townsend coefficient in the following form

$$\alpha = \frac{1}{\lambda_e} e^{-\frac{\beta}{\lambda_e}}, \quad \text{where } \beta = \frac{d\,E_i}{e\,V}. \tag{4}$$

By considering the first Townsend coefficient as a function of the mean free path, $\alpha(\lambda_e)$, and expanding it around a give value λ_e^0

$$\alpha(\lambda) = \alpha(\lambda_e^0)[1 + L_1(\lambda - \lambda_e^0) + L_2(\lambda - \lambda_e^0)^2], \qquad (5)$$

with $L_1 = \frac{1}{\lambda_e^0}(\frac{\beta}{\lambda_e^0} - 1)$ and $L_2 = \frac{1}{2}(\frac{1}{\lambda_e^0})^2[(\frac{\beta}{\lambda_e^0} - 2)^2 - 2]$.

It allows to estimate the strong dependence on mean free path variation. In particular, by substituting all parameters for our experimental setup, d=1cm, E_i=13eV, $\lambda_e^0 = 90$nm, and V=34kV, the values of the first two coefficients are $L_1 \approx 10^6$ and $L_2 \approx 10^{13}$. This result explains strong dependence of the α coefficient on the mean free path variation, shown in Fig. 1c of the main manuscript.

In these expressions, ionization energy, E_i, and second Townsend coefficient, γ, are constants related to the properties of gas and electrodes. Mean free path however depends on local thermodynamics parameters – pressure, p, and temperature, T. Therefore, by controlling it one can control first Townsend coefficient, α, and the breakdown voltage, V_{app}. Assuming that at thermodynamic equilibrium pressure and temperature variations occur at distances that are significantly larger than the man free path (which is a natural assumption as pressure and temperature are macroscopic quantities), we may find an expression for the mean free path assuming that locally (i.e., in a small but macroscopic volume) an ideal gas law is satisfied ($p = nk_B T$, where n is the gas density). By definition:

$$\lambda_e(\mathbf{r}) = (\sigma n(\mathbf{r}))^{-1} = \frac{k_B T(\mathbf{r})}{\sigma p(\mathbf{r})}, \qquad (6)$$

where σ is the scattering cross-section and **r** is the local coordinate. In our case pressure is constant (p=1atm) and only temperature varies.

Using Supplementary Equations (4) and (6) we can find an expression for the first Townsend coefficient as a function of temperature:

$$\alpha(\mathbf{r}) = \frac{\sigma p}{k_B T(\mathbf{r})} \exp\left(-\frac{E_i\, \sigma p d}{eVk_B\, T(\mathbf{r})}\right) \qquad (7)$$

By using the axial symmetry of the problem, for a nonuniform Townsend coefficient, $\alpha(\mathbf{r})$, the Townsend breakdown condition may be found as:

$$\int_0^d \alpha(z)dz = \log\left(\frac{1}{\gamma}\right), \qquad (8)$$

where we $\int dz$ corresponds to the integration along the path that maximizes breakdown probability, i.e., along the temperature gradient.

By knowing the temperature distribution $T(\mathbf{r})$ and second Townsend coefficient, γ, we solve Supplementary Equation (8) for the applied voltage V, that gives a breakdown voltage, $V = V_b$. To find the value of the second Townsend coefficient we study breakdown condition for a regular parallel plate capacitor under normal conditions. In this case, from Supplementary Equations (2) and (7) we obtain:

$$\gamma \simeq \exp(-\alpha_0 d), \ \ with\ \alpha_0 = \frac{\sigma p}{k_B T_0} \exp\left(-\frac{E_i\, \sigma p d}{eV_{b0} k_B\, T_0}\right) = pA \exp\left(-\frac{Bpd}{V_{b0}}\right), \qquad (9)$$

where A and B are constant for a given gas (A=112.5 (kPa·cm)$^{-1}$ and B=2737.5 V (kPa·cm)$^{-1}$ at room temperature T=300K). V_{b0} is the breakdown voltage under normal conditions for a given distance between electrodes d. In our experiments we apply voltage $V = 32kV$ and find distance d at which breakdown occurs. We compare these values with table data for air to ensure that our experiment yields a correct result. We then use Supplementary Equation 9 to find coefficient γ.

For completeness, we extended the calculations for a case of a single particle up to 2000K, which roughly corresponds to the thermal ionization threshold. The results shown in

Supplementary Figure 3 indicate that voltage breakdown can be reduced up 3.5 times for various electrodes separations.

Supplementary Note 2. **TEMPERATURE DISTRIBUTION OF AIR AROUND A HOT PARTICLE**

In general, we are dealing with a non-uniform temperature distribution of air around the hot particle. For simplicity, we consider a single spherical particle with the uniform surface temperature. We also assume that the particle is trapped in the vortex beam for sufficiently long to be in thermodynamic equilibrium. Then the distance-dependent temperature profile outside the particle can be readily approximated as

$$\mathrm{T}(r) = \frac{R(\mathrm{T_s}-\mathrm{T_0})}{r} + \mathrm{T_0} \qquad (10)$$

where R is the particle radius, T_s is the surface temperature, determined experimentally, and T_0 is the ambient temperature. The temperature distribution for several particles was simulated numerically by using Lumerical HEAT solver.

Supplementary Note 3. **ELECTRICAL DISCHARGE GUIDING WITH MULTIPLE TRAPPED PARTICLES**

The ability to trap multiple particles between the electrodes creates additional conditions for a discharge guidance along substantially longer paths and channelling the electrical discharge along the particle positions. Supplementary Figures 2 a-c detail the mechanism for discharge channel formation with multiple particles trapped between electrodes. If the particles size is smaller than the diameter of the channel in a tractor beam, multiple particles trapped in the beam are heated to approximately the same temperature, T_s. The heated particles create a hot channel – a virtual "tube" in mid-air – with an elevated average temperature and longer electron mean free path. The uniformity of the channel depends on the interparticle spacing (Supplementary Figure 2 a and b), and the size the channel is approximately comparable with the tractor beam diameter and is defined by the mean lateral temperature profile. This channel creates a favourable path along which the discharge is guided. Supplementary Figure 2 c shows the calculated maximum distance at which breakdown is observed for multiple trapped particles with various temperatures and interparticle spacing values. Clearly, for a given voltage as the number of particles injected between the electrodes increases the maximum distance of the discharge also grows substantially.

SUPPLEMENTARY REFERENCES

1. Vaill, J. R., Tidman, D. A., Wilkerson, T. D., & Koopman, D. W. Propagation of high-voltage streamers along laser-induced ionisation trails. *Appl. Phys. Lett.* **17**, 20-22 (1970).
2. Guenther, A. H., & Bettis, J. R. A review of laser-triggered switching. *Proc. IEEE*, **59** (4), 689-697 (1971).
3. Koopman, D. W. and Wilkerson, T. D. Channelling of an ionising electrical streamer by a laser beam. *J. Appl. Phys.* **42**, 1883–1886 (1971).
4. Ball, L. M. The laser lightning rod system: thunderstorm domestication. *Appl. Opt.* **13**, 2292–2296 (1974).
5. Aihara, Y. and Shindo, T. Development of long gap discharges guided by a pulsed CO2 laser. *J. Phys. D. Appl. Phys.* **26**, 1244–1252 (1993).

6. Wang, D. et al. A possible way to trigger lightning using a laser. *J. Atmospheric Sol.-Terr. Phys* **57**, 459-466 (1995).
7. Miki, M., Shindo, T., & Aihara, Y. Mechanisms of guiding ability of laser-produced plasmas on pulsed discharges. *J. Phys. D: Appl. Phys.* **29**, 1984 (1996).
8. Polynkin, P. et al. Channeling the dielectric breakdown of air by a sequence of laser-generated plasma filaments. *J. Opt. Soc. Am. B*, **36**, 3024-3029 (2019).
9. Comtois, D. et al. Triggering and guiding leader discharges using a plasma channel created by an ultrashort laser pulse. *Appl. Phys. Lett.* **76**, 819-821 (2000).
10. Tzortzakis, S. et al. Femtosecond laser-guided electric discharge in air. *Phys. Rev. E* **64,** 057401 (2001).
11. Rodríguez, M. et al. Triggering and guiding megavolt discharges by use of laser-induced ionised filaments., *Opt. Lett*. **27**, 772 (2002).
12. Scheller, M., Born, N., Cheng, W., & Polynkin, P. Channeling the electrical breakdown of air by optically heated plasma filaments. *Optica* **1**, 125-128 (2014).
13. Clerici, M., Hu, Y., Lassonde, P., Milián, C., Couairon, A., Christodoulides, D. N., ... & Faccio, D. Laser-assisted guiding of electric discharges around objects. *Science advances* **1**, e1400111 (2015).
