# Supplementary material for: Optical beaming of electrical discharges
Source: Nat Commun. 2020 Oct 20;11:5306. doi: 10.1038/s41467-020-19183-0 (PMC7576779; doi:10.1038/s41467-020-19183-0)
Supplement: Supplementary file 2 — Description of Additional Supplementary Files [file 41467_2020_19183_MOESM2_ESM.pdf]

Description of Additional Supplementary Files

File name: Supplementary Movie 1
Guiding an electrical discharge with a fixed dielectric microsphere
Description: This video shows guiding of electric discharge along trajectory passing through a laser heated dielectric sphere fixed on a post. The distance between the electrodes is 10 mm, applied voltage is 32 kV (i.e., close to breakdown at normal conditions), and the power of the laser beam illuminating the sphere is 500 mW. Initially the laser is off and discharge takes place along random trajectories. However, the discharge passes through the sphere when the particle is heated upon illumination (i.e., with laser on).

File name: Supplementary Movie 2
Guiding an electrical discharge with a fixed metallic microsphere
Description: This video shows guiding of electric discharge along trajectory passing through a laser heated fixed stainless sphere coated with 300 nm carbon layer. The distance between the electrodes is 10 mm, applied voltage is 32 kV (i.e., close to breakdown at normal conditions), and the laser beam power is 1000 mW. Here, again discharge passes through the sphere upon laser illumination.

File name: Supplementary Movie 3
Sub-threshold triggering of an electrical discharge with a fixed laser-heated microsphere.
Description: This video demonstrates triggering of an electrical discharge with the laser-heated microsphere for a subthreshold case, that is, when the distance between the electrodes is longer than that for the breakdown at normal conditions. Here the distance between the electrodes is 12 mm, an applied voltage is 32 kV, and the power of laser beam illuminating the sphere is 500 mW. The laser is illuminating the sphere all the time. The triggering and subsequent guiding of discharge through the sphere occurs when the sphere's temperature reaches 150^0C.

File name: Supplementary Movie 4
Guiding an electrical discharge with a hot metallic microsphere
Description: This video provides experimental evidence that the effect of the guided electrical discharge is not caused by electron photoemission upon laser illumination. Heated metallic microsphere stimulates breakdown and guidance of an electrical discharge even after the laser beam is blocked. The distance between electrodes is 10 mm and the applied voltage is 32 kV.

File name: Supplementary Movie 5
Guiding an electrical discharge with a hot dielectric microsphere
Description: This video provides experimental evidence that the effect of the guided electrical discharge is independent from the electrical properties of the microsphere. The hot glass microsphere stimulates the breakdown and guidance of an electrical discharge even after the laser beam is blocked. The distance between the electrodes is 10 mm and an applied voltage is 32 kV.

File name: Supplementary Movie 6
Formation of a thermal channel in air by the trapped graphene particles

Description: This video shows tractor beam trapping and transport of particles to a space between two planar electrodes. Upon particles' heating a channel with favourable conditions for discharge guidance is created. The discharge does take place but is not visible because of high light scattering from the particles. Applied voltage is 32 kV.

File name: Supplementary Movie 7
Example of an electrical discharge in an empty air
Description: This video shows an electrical discharge in the absence of particles between the electrodes (10 mm apart, 32 kV). The discharge occurs along random trajectories originating and terminating predominantly at electrode edges.

File name: Supplementary Movie 8
Triggering an electrical discharge by particles guided with a vortex beam
Description: This video shows stimulation of an electrical discharge by a chain of heated graphene particles trapped and guided in the vortex laser beam. Discharge is not visible because of high light scattering form the particles. When the supply of the particles is interrupted (as seen on the left of the frame due to an electrical discharge in the vertical cuvette), the discharge is no longer guided and, instead, follows random trajectories close to the edges of electrodes. The distance between planar electrodes is 10mm and the applied voltage is 32 kV.

File name: Supplementary Movie 9
Electrical discharge stimulated by particles trapped with a vortex beam
Description: This video demonstrate the electrical discharge stimulated by particles trapped and heated in the tractor laser beam. The initial visible discharge occurs along the trajectory connecting the particles trapped in the beam. For some time after that, although there are no more particles transported by the beam, some discharges still travel close to the beam because of remnants of the hot air channel created by initial discharge. As the temperature in the channel gets back to normal condition discharges follow random trajectories. The distance between electrodes is 10mm and the applied voltage is 32 kV.

File name: Supplementary Movie 10
Long range triggering and guidance of electrical discharge with a laser beam.
Description: This video shows the evolution of the electrical discharge in the case of multiple particles trapping. The electrical sparks propagate through the 30 mm gap along trajectory connecting all particles trapped inside the tractor beam. The voltage applied (32 kV) is well below the threshold for the air breakdown at 1 atm for the 30 mm separation distance.
